# Long-term persistence and effects of fetal microchimerisms on disease onset and status in a cohort of women with rheumatoid arthritis and systemic lupus erythematosus

**DOI:** 10.1186/1471-2474-14-325

**Published:** 2013-11-18

**Authors:** Marianne Kekow, Maria Barleben, Susanne Drynda, Sibylle Jakubiczka, Jörn Kekow, Thomas Brune

**Affiliations:** 1 Children's Hospital, University of Magdeburg, Leipziger Str. 44, Magdeburg 39120, Germany; 2 Clinic of Rheumatology, University of Magdeburg, Sophie-von-Boetticher-Strasse 1, Vogelsang, Gommern 39245, Germany; 3 Institute of Human Genetics, University of Magdeburg, Leipziger Str. 44, Magdeburg 39120, Germany

**Keywords:** Microchimerism, RA, SLE, Pregnancy

## Abstract

**Background:**

The discovery of a fetal cells transfer to the mother is a phenomenon with multiple implications for autoimmunity and tolerance. The prevalence and meaning of the feto-maternal microchimerism (MC) in rheumatic diseases has not been thoroughly investigated. The aim of this study was to analyze the prevalence of fetal MC in patients with inflammatory rheumatic diseases and to investigate the association of MC with disease onset and current status.

**Methods:**

A total of 142 women who gave birth to at least one male offspring were recruited: 72 women with rheumatoid arthritis (RA), 16 women with systemic lupus erythematosus (SLE), and 54 healthy women. For the detection of fetal microchimerism a nested PCR method was used to amplify a Y chromosome specific sequence (TSPY1). For characterization of disease activity we analyzed autoantibody profiles and X-rays in RA, and in addition complement levels in SLE respectively.

**Results:**

A significant higher prevalence of fetal MC was found in RA (18%) and SLE (31%) compared to controls (3.7%) (p = 0.02 and p = 0.006, resp.). The mean age at disease onset was comparable in MC + and MC- RA patients. Disease onset occurred 18.7 (MC +) and 19.8 (MC-) years post partum of the first son, respectively. The presence of anti-CCP and RF did not differ significantly, anti-CCP were found in 75% of MC + and 87% of MC- patients, RF in 75% of both MC + and MC- patients. A slightly higher mean Steinbrocker score in MC + patients was associated with longer disease duration in MC + compared to MC- RA. In SLE patients the mean age at disease onset was 42.6 years in MC + and 49.1 years in MC- patients. Disease onset occurred 24.0 and 26.4 years post partum of the first son for MC + and MC- patients, respectively. The presence of ANA and anti-dsDNA antibodies, C3, C4 and CH50 did not differ significantly.

**Conclusion:**

Our results indicate a higher frequency of long-term male MC in RA and SLE patients compared with controls without impact on disease onset and status in RA and SLE.

## Background

Rheumatoid arthritis (RA) and systemic lupus erythematosus (SLE) are autoimmune diseases with a higher prevalence in women than in men (3:1 women vs. men in RA and 9:1 in SLE, respectively) [1]. The cause and onset of the diseases still remain unclear. RA and SLE are, once established, highly affected by pregnancy. Guthrie et al. reported protective effects of pregnancy for the development of RA in women [2]. In this study the risk of developing RA increased with the number of years after the birth of the youngest child. These findings, together with the first description of fetal DNA in pregnant women [3,4] gave raise to the question of the role of this chimerism for the development of autoimmune diseases. The presence of very small amounts of cells or DNA is now called microchimerism (MC), e.g. detectable by PCR [5,6]. Recently the fetal DNA became a focus of interest as a non invasive diagnostic tool for chromosomal abnormalities such as Down`s syndrome in pregnant women [7,8].

In autoimmune diseases the frequency of MC differs but studies indicate that the frequency of MC is higher in women with some autoimmune diseases than in healthy women [6]. In the field of rheumatic diseases patients with scleroderma, RA, SLE und Sjögren’s syndrome have been investigated [9-16]. However, data are inconsistent due to small numbers of patients, different methodology, and patient selection.

Prevalence studies in healthy women by our group found the TSPY1 gene as MC indicator present in about 70% of the women at delivery declining to 4% after a 4 year follow up [17]. This is quite comparable with other reports [13,18-20]. For the present study we could apply the detection of a TSPY1 gene sequence on the Y chromosome in patients even decades after delivery.

The aim of our study was to evaluate the frequency of microchimerism in RA and SLE patients and to investigate the effects of fetal microchimerisms on disease onset and status. The detection of fetal MC was performed by nested PCR. Other sources for male DNA were strictly excluded by questionnaire. Different clinical measures, X-ray and laboratory parameters were collected from the patient’s records. The comparison of MC positive and MC negative RA and SLE patients with healthy controls revealed a long persistence of MC.

## Methods

### Patients

All together 142 female subjects were studied: 72 patients with RA, 16 patients with SLE and 54 healthy controls. All subjects were selected based on the following criteria: (*a*) a previous pregnancy with at least one male offspring, (*b*) no history of abortion, and (*c*) no history of blood transfusions. The patients had to meet the American College of Rheumatology criteria for RA and SLE respectively [21,22]. Clinical data on the patients with RA and SLE were collected from their medical records including their X-rays, and laboratory results. Special family history was obtained by questionnaires. The subjects were outpatients in one rheumatology centre (Clinic of Rheumatology, University of Magdeburg).

The study was approved by the local human subjects committee of the University of Magdeburg (approval number 133/04), and all patients were asked for written consent.

For RA patients, the following data were collected: rheumatoid factor (RF); anti-cyclic citrullinated peptide antibodies (anti-CCP); and structural damage as defined by the Steinbrocker score for X-rays of hand and feet [23,24]. All X-rays were blinded and red separately by two rheumatologists. RF and anti-CCP were determined by commercial assays (ABX Pentra RF CP, HORIBA, Germany; and anti-CCP-ELISA, A.Menarini Diagnostics, Italy, respectively).

In SLE patients, the antinuclear antibodies (ANA), the dsDNA antibodies (dsDNA Ab), serum C3, C4, and CH50 were analyzed. ANA testing was performed by indirect immune fluorescence, and the determination of dsDNA Ab by ELISA (all Euroimmun AG, Germany). Determination of complement factors C3, C4 and CH50 was run using a turbidimetric method with assays from Biokit, Spain (C3, C4), and Wako Chemicals, Germany (CH50).

### Sample preparation and nested PCR

Genomic DNA from peripheral blood was extracted using the QIAamp® DNA Blood Midi Kit (QIAGEN, Germany) according to the manufacturer’s instructions. Blood samples from a male subject and a nullipara woman were used as positive and negative controls.

A sequence in the TSPY gene located on the short arm of the Y-chromosome (encoding the testis specific protein Y-linked 1, and also known as CT78 and DYS14) was detected by amplifying genomic DNA in a nested polymerase chain reaction (PCR). Primers were designed based on a sequence described by Arnemann et al. [25]. The following primer pairs were used for the first and second PCR reactions: first PCR: forward: 5´- ATG CGG CAG AGA AAC CCT TG - 3´; reverse: 5´- TAA GGC CTC CTG TGT TCA CG - 3´ and second PCR: forward: 5´- CAG AAG CGA GTT CAG AGC AG - 3´; reverse: 5´- TTC TGA GGC TGA CTG CAC TG - 3´.

The first PCR reaction was performed in a total volume of 50 μl and contained 5 μl genomic DNA at a concentration of 80-100 μg/ml, 5 μl 10 fold Taq polymerase buffer, MgCl_2_ at a final concentration of 50 mM, 20 pmol of each primer, 4 μl of the dNTP mix (2 mM total) and 1.5 units of Taq DNA polymerase (Invitrogen, USA). PCR conditions were as follows; initial denaturation at 94°C for 5 minutes, 35 cycles at 94°C for 60 seconds; 63°C for 60 seconds; and 72°C for 60 seconds; and a final extension at 72°C for 10 minutes. Two μl of the reaction mixture of the first reaction containing the 278 bp amplificate were used as template in the second PCR reaction in a total volume of 20 μl. The second primer pair was used, beyond that the composition of the reaction mixture was identical to the first reaction. The PCR conditions were also similar to the first reaction, but only 25 cycles were run. The PCR products were separated on a 2% agarose gel in 1fold TRIS borate-EDTA buffer. For sizing the 186 bp product a 100 bp ladder was used.

Each PCR amplification step included a positive control (DNA from a male donor, diluted 1:20,000 in water for molecular biology), and a negative control (DNA-free water) to detect PCR contamination. All samples were tested twice.

The PCR was optimized to detect male-specific DNA sequences in DNA preparations from a mixture of blood from a male and a female donor at a ratio of 1:40,000. A reliable detection of male blood in female blood, male DNA in female DNA and male DNA in Tris-EDTA buffer with a mixture ratio of 1: 40,000 was possible with the optimized PCR.

### Data processing and analysis

For analysis, the software package SPSS was used (V 18.0). Data are presented as mean, standard error of the mean (SEM), min-max, and the 95% confidence interval. The t-test was used to determine the significance of differences between the means of independent samples. Differences in frequencies were analysed by applying the Fisher’s exact test.

## Results

The overall prevalence of fetal MC was 18.1% in RA patients (mean 37.3 years after the birth of the last son) and 31.3% in patients with SLE (mean 29.8 years after the birth of the last son) which is significantly higher as compared to 3.7% in healthy controls (HC) (p = 0.023 and p = 0.006, resp.) (Table 1).

**Table 1:** Overall prevalence of fetal MC (HC = healthy controls)

	**RA**^**1**^	**SLE**^**2,3**^	**HC**
MC +	13 (18.1%)	5 (31.3%)	2 (3.7%)
MC -	59 (81.9%)	11 (68.7%)	52 (96.3%)

^1^RA vs. HC: p = 0.023, ^2^SLE vs. HC: p = 0.006, ^3^RA vs. SLE: p = 0.303.

In healthy controls, the mean age at the birth of the first son was 25.9 years which is comparable to the RA and SLE patients. None of our patients or controls had disabled children.

### Rheumatoid arthritis patients

In RA patients the disease onset was 18.7 and 19.8 years post partum of the first son for MC + and MC- patients, respectively (Table 2). Patients with two or more sons had a higher frequency of MC than patients with only one son without reaching statistical significance (p = 0.218) (Table 3). The presence and the levels of anti-CCP and RF did not differ significantly: anti-CCP were found in 75% of MC + and 87% of MC- RA patients; RF in 75% of both MC + and MC- patients (Table 2).

**Table 2:** Patient characteristics of MC + and MC- RA patients at study entry

	**MC + RA**	**MC- RA**	**all RA**
Mean age at study entry (SEM)	66.0* (2.5)	61.2* (1.4)	61.7 (1.3)
min-max	48-76	41-84	41-84
95% CI of SEM	58.6/68.5	58.3/64.1	59.2/64.3
Mean age at disease onset (SEM)	43.3* (3.2)	43.3* (1.7)	43.3 (1.5)
min-max	24-59	18-72	18-72
95% CI of SEM	36.1/50.4	40.0/46.7	40.4/50.4
Mean duration (years) between birth of first son and disease onset (SEM)	18.7* (2.9)	19.8* (1.5)	19.6 (1.3)
min-max	0-29	0-37	0-37
95% CI of SEM	12.3/25.5	17.0/22.8	12.3/25.5
Mean age at birth of first son (SEM)	25.4* (1.5)	23.4* (0.6)	23.7 (0.6)
min-max	20-35	18-38	18-38
95% CI of SEM	22.0/28.8	22.9/24.6	22.0/28.8
CCP-antibody level	**n =**	**n =**	**n =**
0-25 IU/ml (negative)	3 (25%)	7 (13%)	10 (15%)
26-100 IU/ml (positive)	1 (8%)	9 (17%)	10 (15%)
101-1000 IU/ml (positive)	5 (42%)	23 (43%)	28 (42%)
>1000 IU/ml (positive)	3 (25%)	15 (28%)	18 (27%)
Total CCP-antibody positive	9* (75%)	47* (87%)	56 (84%)
RF level: 0-20 IU/ml (negative)	3 (25%)	13 (24%)	16 (24%)
21-100 IU/ml (positive)	3 (25%)	31 (56%)	34 (51%)
>100 IU/ml (positive)	6 (50%)	11 (20%)	17 (25%)
Total RF positive	9* (75%)	42* (75%)	51 (75%)
Patients treated with ‘Biologicals’	10 (77%)*	40 (68%)*	50 (70%)
Steinbrocker score 1	1 (8%)	6 (10%)	7 (10%)
2	2 (15%)	11 (22%)	13 (20%)
3	3 (23%)	16 (30%)	19 (29%)
4	7 (53%)	22 (37%)	29 (41%)
Mean Steinbrocker score	3.23*	3.04*	3.07

*p (MC + vs. MC-) not statistically significant.

**Table 3:** Number of sons in RA and SLE patients

	**1 son**	**≥2 sons**
^1^RA patients MC+	7 (63.6%)	4 (36.2%)
^1^RA patients MC-	47 (82.5%)	10 (17.5%)
Total RA patients	54 (79.4%)	14 (20.6%)
^2^SLE patients MC+	3 (60%)	2 (40%)
^2^SLE patients MC-	10 (91%)	1 (9%)
Total SLE patients	13 (81%)	3 (19%)

^1^RA: number of sons MC + vs. MC-: p = 0.218.

^2^SLE: number of sons MC + vs. MC-: p = 0.214.

A slightly higher Steinbrocker score in MC + patients (3.2 vs. 3.0) was associated with longer disease duration of about 23 years in MC + RA and 18 years in MC- RA with no evidence for structural damage due to MC positivity (Table 2).

The frequency of biological therapies, mostly TNF blocking agents, was comparable in both groups (Table 2). More than 80% of the patients received methotrexate at a dosage of 15 to 25 mg / week, and took steroids up to 7.5 mg per day. Due to the limited number of patients, conclusions may be difficult.

### Systemic lupus erythematosus patients

In SLE, the mean age at disease onset was 42.6 years in MC + and 49.1 years in MC- patients. Disease onset occurred 24.0 and 26.4 years post partum of the first son for MC + and MC- patients, respectively (Table 4). Thirteen out of 16 patients with SLE (81%) have given birth to only one son. 40% of MC + patients have more than one son compared to only 9% of MC- patients (Table 3). The presence of ANA and dsDNA antibodies, and the lab test results including C3, C4 and CH50 did not differ significantly (Table 4). In MC + patients sicca symptoms were found most frequently (80%) followed by arthritis (60%), central nervous system abnormalities (20%) and kidney involvement (20%). In contrast, in MC- patients joints were affected in 70% of the cases followed by skin (60%), central nervous system (30%) and sicca symptoms (20%).

**Table 4:** Patient characteristics of MC + and MC- SLE patients at study entry

	**MC + SLE**	**MC- SLE**	**all SLE**
Mean age (SEM)	51.2* (3.4)	57.1* (3.7)	55.2 (2.7)
min-max	42-58	45-82	42-82
95% CI of SEM	41.6/60.8	48.9/65.5	49.8/60.7
Mean age at disease onset (SEM)	42.6 *(3.8)	49.1* (4.5)	46.9 (3.3)
min-max	35-54	27-75	27-75
95% CI of SEM	32.1/53.1	39.0/59.2	40.0/54.0
Mean age at birth of first son (SEM)	24.0* (2.4)	26.4* (1.6)	24.7 (1.3)
min-max	18-31	22-38	18-38
95% CI of SEM	16.3/31.7	22.7/30.1	22.8/28.7
ANA titer (Median)	1:640	1:640	1:640
(n = 10 MC- n = 4 MC+)			
min-max	160-2560	160-2560	160-2560
dsDNA-antibodies present	0 / 3 (0%)	3 / 10 (30%)	3 / 13 (23%)
C3 (g/l) Mean (n = 9 MC-, n = 2 MC+)	1.47	1.28	1.32
min-max	1.45-1.50	0.94-1.55	0.94-1.55
C4 (g/l) Mean (n = 9 MC-, n = 2 MC+)	0.28	0.23	0.24
min-max	0.22-0.35	0.13-0.33	0.13-0.35
CH50 Mean (U/ml)	56.8	51.4	52.3
(n = 9 MC-, n = 2 MC+) min-max	52.7-60.9	42.1-59.1	42.1-60.9

Normal ranges: C3 0.9-1.8 g/l; C4 0.1-0.4 g/l; CH50 23–46 U/ml; ANA: 1:80–1:320 (+ - ++), 1:640–1:1280 (+++), >1:2560 (++++); dsDNA Ab: < 100 RE/ml; *p (MC + vs. MC-) not statistically significant.

## Discussion

Microchimerisms have been investigated for more than 30 years with different methods. With sensitive techniques such as PCR even very small amounts of male DNA can be detected in the female organism [26-28]. In the present study, patients with RA and SLE were tested for the occurrence of the TSPY1 gene in the peripheral blood. The share of women with MC in RA patients was significantly increased compared to the control group. The prevalence of a MC in SLE patients was also significantly increased compared to the control group. Compared with the RA group, the prevalence is increased in the SLE group, but does not achieve statistical significance, which may be due to the limited number of patients.

A comparison of our data on the prevalence of MC with other reports has limitations. The detection methods used in each study and the experimental setting differ notably (Table 5) [14,29-37]. In addition, different inclusion and exclusion criteria (especially the inclusion of blood transfusions or abortions) can affect the frequency of detected MC. Depending on methods and patients selected, the prevalence of MC in RA patients can be found between 18% (Yan et al.) and 42% (Rak et al.) [14,30]. The study of Yan et al. compared the frequency of MC in 71 patients with RA and 49 healthy controls without analyzing the severity of disease or duration of disease. Women with a history of abortion were not excluded from the study. In healthy individuals they found a slightly elevated frequency of MC (24%) compared to RA patients (18%) [31]. Studying 25 pregnant RA patients the same group reported a correlation of RA disease activity and fetal DNA levels in pregnant women [30]. Rak et al. used the detection of fetal HLA-DRB1 sequences in the maternal blood for the detection of MC transmitted to the mother [14]. However, blood transfusions and early fetal loss were not excluded. They speculate that MC may contribute to the risk of an autoimmune disease by providing susceptibility alleles. Using the disease activity score 28 (DAS28) no correlation with the presence of MC was found [14]. Time between the last pregnancy and the DNA testing was not provided making a direct comparison with our data difficult. Atkins et al. tested tissue from rheumatic nodules of 15 RA patients for male DNA using a PCR for the DYS14 sequence [32]. 74% of the removed rheumatic nodules from RA patients tested positive for male DNA. They found no association of MC and joint destruction [32]. Besides peripheral blood and nodules, other tissues were also investigated for MC. Hromadnikova et al. analyzed synovial tissue and skin samples of 19 RA patients for the SRY gene [34]. The patients had a mean age of 55.2 years and had given birth to at least one son. They compared the results to samples from RA patients without sons. The synovial tissue of 5 out of 13 women (38.5%) and 4 out of 10 skin samples contained male DNA. In samples from patients without male offspring, no male DNA was found [34].

**Table 5:** Overview of major MC studies in RA and SLE patients

**Disease**	**Number of subjects/samples**	**Method/ Material**	**MC positive**	**Mean disease duration (years)/age of subjects (years)**	**History of blood transfusion**	**References**
RA	71 RA; 49 HC	PCR/ PBMC	18% RA, 24% HC	n.r. / 33 (median)	n.r.	Yan et al. [31]
RA, JIA	25 pregnant patients:	PCR/ PBMC		n.r. / 33	n.r.	Yan at al. [30]
	(21 with improvement of arthritis, 4 with active arthritis)		100%50%			
RA	DRB1*01 MC:	PCR/PBMC	DRB1*01 MC:	n.r./ 57 (RA);	included	Rak et al. [14]
	33 RA, 46 HC		RA: 30%, HC: 4%	52 (HC)	(17%, HC 13%)	
	DRB1*04 MC:		RA: 40%,			
	48 RA, 64 HC		RA: 40%, HC: 8%			
RA	15 patients/19 granulomatous nodules	PCR/ tissue	13/15 patients, 14/19 nodules	22.5 / 66	included	Atkins et al. [32]
RA	QKRAA MC:	PCR/ PBMC	QKRAA MC	n.r. / 51 (RA),	n.r.	Yan et al. [29]
	52 RA, 34 HC		RA: 17%, HC: 3%	42 (HC)		
	QRRAA MC:		QRRAA MC:			
	52 RA, 34 HC		RA: 40%, HC: 18%			
RA	13 patients (synovial tissue), 10 patients (skin fibroblasts)	PCR/tissue	38%	16.4 / 55.5	n.r.	Hromadnikova et al. [34]
			40%			
SLE	1 patient, 44 samples, 11 female control subjects	FISH/tissue	100% abnormal tissue, 0% normal tissue; 0/11 controls	1 / 33	n.r.	Johnson et al. [33]
SLE	22 patients, 24 HC	PCR/whole blood	SLE: 50% HC: 50%	n.r. /44 (SLE), 48 (HC)	excluded	Mosca et al. [35]
SLE	49 patients (57 renal biopsies), 51 HC samples	FISH/tissue	SLE: 55% of patients, 51% of samples HC: 25% of samples	n.r. / 31 (SLE)	included (31%)	Kremer Hovinga et al. [37]
SLE	7 patients (48 organ samples), 34 HC (146 samples)	FISH/tissue	SLE: 100% (50% of samples), HC: 44%, (14% of samples)	7 /41 (SLE); 47 (HC)	excluded	Kremer Hovinga et al. [36]

n.r.: not reported, FISH: fluorescent in situ hybridization. HC: healthy controls.

In SLE patients, varying data for the prevalence of MC can be found as well. The frequencies range between 0% found by Miyashita and 50% as described by Mosca et al. [15,35]. The latter group analyzed blood samples from 22 SLE patients and 24 healthy controls for the Y chromosome using a nested PCR method with strict exclusion criteria. For both groups, in 50% of the blood samples MCs were detected which may be explained by a broad variation time between last pregnancy and the time point of DNA testing. They found no differences in ANA, dsDNA-Ab, and complement levels associated with or without MC [35].

Besides pregnancy, high rates of abortions in SLE may be a major source for feto-maternal MC. A meta-analysis of 11 studies by Khosrotehrani et al. could identify a higher rate of MC in patients with fetal loss, which was independent from the number of gestations [38]. Tissue was often subject for studies of MC in SLE [36,37]. MC was found in different tissues including kidney, intestine or lung [33].

In our study, patients who had received blood transfusions were excluded. This is because the kinetic of the removal of foreign cells and DNA is not yet fully understood [39]. In addition, it is unknown whether the blood transfusion is from a male or female donor. Even if the average percentage of male blood donors is known, it is difficult to calculate its influence on the prevalence of MC. By excluding RA and SLE patients with blood transfusions in the past more than 50% of the potential candidates for our study were excluded. A reason for this high number could be a higher frequency in joint replacement surgery, cardiovascular diseases, and anemia by the disease itself or due to side effects of drugs. Many of the investigated patients reported blood transfers in conjunction with gynecological surgery.

Another issue which has to be taken into account is that all of our RA and SLE patients were treated with anti-inflammatory and immune modulating medication. The treatment reduces the progression of the disease and decreases differences between treated and non-treated patients. For selecting RA patients, we used a large local database including many patients receiving biological therapies. Active treatment may have influence on the progression including structural damage and laboratory parameters but this may also have impact on the clearance of fetal components. For RA we chose two serological parameters which are associated with a high disease burden and a poor prognosis (RF and anti-CCP). Both antibodies were found to be positive in a high number of patients, and the absolute values indicate a more severe disease. This is also reflected by the X-ray readings and the high percentage of biological treated patients. Further activity scores like the DAS28 were not applied since they may vary over time. Rak et al. could not find a correlation between the presence of MC and the DAS28 in a small cohort of eight women [14].

For SLE, we looked for single organ manifestations related to the disease. The obtained laboratory data indicate no active disease when the blood was taken for MC analysis. As mentioned before no relationship between the course of the disease and the presence of MC could be detected. Patients with an early stage of disease were not included in this study, so that it would need further investigations including patients with early stages of disease to show an influence of the MC on the disease onset. So far outliers in our study were not associated with MC.

Based on post mortem studies and animal experiments, Kremer Hovinga et al. discussed the role of a MC in patients with SLE in detail [40]. One hypothesis is that chimeric T-lymphocytes can provoke a graft-vs.-host reaction; which means that by the recognition of host cells as foreign, activated chimeric T-helper lymphocytes may even stimulate the production of SLE specific antibodies. Another possible explanation is that the chimeric cell itself is the target of the host-vs.-graft reaction. Antigens present in maternal tissues will stimulate the immune system when the removal of chimeric antigens is poor, e.g. caused by impaired CD8 or natural killer cell function. As in our study, however, a relationship between the presence of chimerism and the clinical picture of the SLE is lacking. In a third hypothesis authors propose a healing effect of the progenitor chimeric cells replacing already damaged tissue. A consequence could be that the new tissue could induce a graft-vs.-host reaction with further tissue damage. Reviewing the literature the authors also underline the difficulty of carrying out such studies in human SLE due to a high rate of unrecognized miscarriages leading to feto-maternal transfer of blood. In addition, animal studies are difficult to transfer into human.

## Conclusion

In conclusion our data indicate a long persistence of MC as a potential marker of pathologic clearance of semi-allogeneic DNA in rheumatic diseases without effects on disease onset and status. MC could be an epiphenomenon of autoimmunity and therapy without having an effect on the clinical manifestation and the phenotype of RA and SLE. Long-term randomized controlled trials, even together with the introduction of new DMARDs, would offer the possibility to detect the role of MC in autoimmune diseases in more detail.

## Abbreviations

ANA: Anti-nuclear antibody; CCP: Cyclic citrullinated peptide; FISH: Fluorescence in situ hybridization; HC: Healthy controls; JIA: Juvenile idiopathic arthritis; MC: Microchimerism; PCR: Polymerase chain reaction; RA: Rheumatoid arthritis; SLE: Systemic lupus erythematosus; TE buffer: Tris-EDTA buffer; TSPY1: Testis specific protein Y-linked 1; yrs: Years.

## Competing interests

The authors declare that they have no competing interests.

## Authors’ contributions

MK was involved in sample and data collection, carried out the genetic analysis and interpretation of data and drafted the manuscript. MK had full access to all of the data in the study and takes responsibility for the data and the accuracy of the data analysis. MB was involved in sample collection and preparation, and acquisition of data. SD contributed to patients’ selection, data acquisition, interpretation and manuscript preparation. SJ contributed to the assay design. JK contributed to the concept and design of the study. TB conceived the study and was involved in developing the concept and design of the study and coordination. All authors substantially contributed to the study and approved the final manuscript.

## Pre-publication history

The pre-publication history for this paper can be accessed here:

http://www.biomedcentral.com/1471-2474/14/325/prepub
